# Allosteric modulation of peptide ligand binding to Neuropeptide Y receptor Y1 revealed by fluorescence-based assay

**DOI:** 10.1186/2193-1801-4-S1-P52

**Published:** 2015-06-12

**Authors:** Santa Veiksina, Sergei Kopanchuk, Ago Rinken

**Affiliations:** Institute of Chemistry, University of Tartu, Estonia; Competence Centre on Health Technologies, Estonia

**Keywords:** Allostery, Fluorescence assay, Neuropeptide Y receptor Y1

Implementation of fluorescence methods in studies of ligand binding to their receptors opens new possibilities to characterise these processes. One of the potential approaches is the detection of changes of the fluorescence anisotropy (FA) and/or total fluorescence intensity (TFI) signals upon binding reaction. However, to achieve significant changes in the FA/TFI signal, some requirements need to be met – the concentration of receptor binding sites as well as the dissociation constant of the interaction should be in the same order as the fluorescent ligand’s concentration. We have used FA assay to investigate ligand binding properties to Melanocortin 4 (MC4) receptor [1]. Implementation of budded baculoviruses (BBV) that display G protein-coupled receptors on their surfaces significantly increased sensitivity and temporal stability of this assay [2]. For the first time we demonstrate the applicability of BBV experimental setup to study Y1 receptor system. Here we used TAMRA-PYY, an Y1 receptor specific fluorescent peptide ligand, as a reporter ligand. Besides real-time monitoring of FA signal changes, up to 5 fold decrease in TFI signal was observed within TAMRA-PYY binding to the Y1 receptor. Pharmacological characterization of Y1 receptors with receptor-specific unlabelled ligands gave the rank order of potencies consistent with previously reported values. Additionally, allosteric heterogeneous interactions were revealed as koff values of TAMRA-PYY differed more than 7 times depending on the nature of dissociation initiated ligand. These observations provide evidence for similar allosteric receptor-ligand binding mechanism as previously shown for MC4 receptors [3].
